# The Genetic Diversity of the Asian Spongy Moth, *Lymantria dispar asiatica* Vnukovskii (Lepidoptera: Erebidae), in Korea Based on Mitochondrial COI Analysis

**DOI:** 10.3390/insects16090958

**Published:** 2025-09-11

**Authors:** Jongmin Bae, Hye-Min Byun, Subin Choi, Geunho Jang, Minjoon Kang, Eunji Kim, Jaekook Park, Heung-Sik Lee, Sunghoon Jung

**Affiliations:** 1 Laboratory of Systematic Entomology, Department of Applied Biology, College of Agriculture and Life Sciences, Chungnam National University, Daejeon 34134, Republic of Korea; jemby5417@gmail.com (J.B.); torhmb@gmail.com (H.-M.B.); subin9319@gmail.com (S.C.); geunho1026@gmail.com (G.J.); rkdalswns4@naver.com (M.K.); eunji95k@gmail.com (E.K.); delphacids@gmail.com (J.P.); 2 Department of Smart Agriculture Systems, College of Agriculture and Life Science, Chungnam National University, Daejeon 34134, Republic of Korea; 3 Plant Quarantine Technology Center, Department of Plant Quarantine, Animal and Plant Quarantine Agency, Gimcheon-si 39660, Gyeongsangbuk-do, Republic of Korea

**Keywords:** gypsy moth, spongy moth, haplotype network, the Korean Peninsula, population genetics, COI gene

## Abstract

In this study, we assessed the genetic diversity of the Asian spongy moth, *Lymantria dispar asiatica* Vnukovskii (Lepidoptera: Erebidae), in Korea. We obtained 123 sequences of the species, including those from 26 regions in Korea. We analyzed the genetic diversity within the Korean samples (*n* = 87) and haplotype networks between the Korean and global samples (123 sequences from 26 regions in Korea and five other countries) using median-joining (MJ) network analysis. The results showed low nucleotide diversity and high haplotype diversity (*π* = 0.00159; *Hd* = 0.660). The neutrality tests were also significantly negative. The MJ network recovered a star-shaped network with diverse populations in Korea, with 12 haplotypes and a dominating haplotype, H07, in all regions. Two haplogroups, Middle and Southern, were identified. The Middle haplogroup included haplotypes from Shandong, China, indicating shared populations between the two regions. In contrast, the Southern haplogroup, primarily found in ship and harbor samples, likely originated from invasive populations. This pattern reflects the influence of human activities such as international trade, highlighting the importance of strict monitoring at ports to prevent the introduction of invasive pests and to support effective forest pest management.

## Simple Summary

The Asian spongy moth is one of the most severe forest pests, having experienced dramatic outbreaks that have led to severe defoliation in Korea. We assessed the genetic diversity of this species. We collected 123 sequences from Korea and adjacent countries, and the collecting locations cover the whole distribution of the Asian spongy moth in Korea. Our results revealed low nucleotide diversity and high haplotype diversity within Korea. Two haplogroups, the Middle and Southern haplogroups, were identified in Korea. Their genetic relationships with other haplotypes and their geographic distribution suggest that Korean populations have been influenced by human activities such as international trade.

## 1. Introduction

*Lymantria dispar* (Lepidoptera: Erebidae) is a polyphagous insect that feeds on more than 100 families of deciduous trees and conifers [1,2,3]. Infested trees suffer severe defoliation and, in some cases, may die [2]. Due to its broad host range and voracious feeding habits, an outbreak of the Asian spongy moth, *Lymantria dispar asiatica* Vnukovskij, in 2020 caused severe damage to forests in the Korean Peninsula [4]. In addition, the European subspecies, *Lymantria dispar dispar* (Linnaeus), has accidentally invaded non-native regions, such as North America [5,6,7,8]. The introduced population has become a serious pest, and this species has been listed among the world’s most severe alien species [9].

Because of the ecological and economic impacts, numerous studies have focused on understanding its genetic characteristics. George [10] revealed that the Canadian populations originated from France. Bogdanowicz et al. [11] identified four groups using mitochondrial DNA among global populations: Okinawa (Japan), Hokkaido (Japan), the rest of Japan + mainland Asia, and Europe + Tunisia + North America. Keena et al. [12] separated the spongy moth into three groups: North American, European/Siberian, and Asian. deWaard et al. [13] constructed a COI barcode reference library and identified three clades within *Lymantria dispar*: North America + France (*L. d. dispar*), Europe + West Asia (*L. d. dispar*), and East Asia (*L. d. asiatica/japonica*). Qian et al. [14] examined COI gene differences among subspecies, supporting the division into three subspecies. Wu et al. [5] analyzed the genetic structure, identified four distinct clusters (three subspecies and the North American population), and reported increased generic variation in East Asia. Kang et al. [15] focused on populations in Far East Asia, revealing three genetic groups within Eastern Asia (central Korea and adjacent regions, southern Korea, and Hokkaido) and suggesting a mixed genetic pattern linked to expansions during the Würm glacial period. Zhao et al. [16] analyzed Chinese populations and found that none of the three subspecies were monophyletic. They also revealed four clusters within China and an independent Black Sea–Caspian genetic lineage. Zahiri et al. [17] studied the global phylogeography of *Lymantria dispar* using mitochondrial and nuclear genes, identifying three mtDNA lineages, Transcaucasia, East Asia + Japan, and Europe + Central Asia, suggesting the Transcaucasia region as the ancestral area for the entire *dispar* group.

While numerous studies have examined the genetic characteristics of this species, only a few have included Korean populations. Currently, there is no research based on samples collected across South Korea. Given that the Korean Peninsula may serve as a geographic bridge connecting the Japanese archipelago and the Asian continent, understanding the genetic diversity of the Korean population is important. In addition to this geographic role, the Korean population has expanded and dispersed beyond its original habitats due to recent outbreaks. Moreover, due to their habit of ovipositing on various substrates, such as tree trunks and cargo, spongy moth egg masses are easily transported internationally via cargo ships and other vessels. Invasive populations from other countries have been detected on vessels during quarantine inspections, suggesting the possibility of interactions between native and alien populations. Therefore, we expect that both the outbreak and introductions of alien populations have affected the genetic diversity of this species in Korea. In this study, we aim to characterize the genetic diversity of the Korean population and to examine the relationships between the Korean and global populations. This assessment is essential for understanding population relationships, monitoring the effects of recent outbreaks, and informing management strategies to prevent further spread.

## 2. Materials and Methods

**Figure 1:**
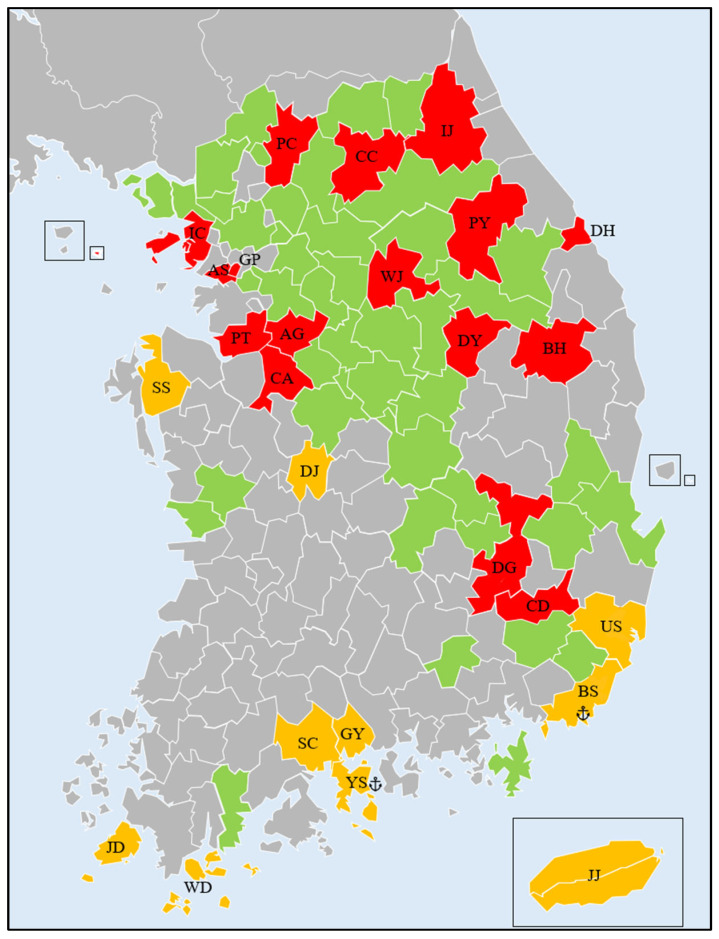
Sampling locations in Korea. The green areas represent the known distribution of *Lymantria dispar asiatica*, as reported by Jung et al. [20]. The red colors indicate sampling locations within this known distribution range. The orange colors show sampling locations outside the previously reported distribution. Anchor marks indicate harbors.

We obtained COI sequences of *L. dispar asiatica* from 17 different regions in Korea and also downloaded them from GenBank (NCBI) (Table 1 and Figure 1). To minimize sampling bias, no more than five sequences from the same locality were included. The origins of ship-collected samples were verified by the Animal and Plant Quarantine Agency, Korea (APQA). In total, 123 sequences from 26 regions in Korea and five other countries were analyzed.

DNA extraction was performed using the QIAamp DNA Mini Kit (Qiagen, Hilden, Germany), and PCR was conducted using Solg 2X Taq PCR Pre-mix (SolGent Co., Ltd., Daejeon, Republic of Korea) with the primer set LepF1/LepR1 [21] to amplify the cytochrome oxidase I (COI) gene. PCR products were sequenced by Macrogen Inc. (Seoul, Republic of Korea), and all sequences were submitted to NCBI GenBank (Supplementary Table S1).

Indices of genetic diversity and neutrality statistics (Tajima’s *D*, Fu and Li’s D*, and Fu and Li’s F*) were calculated using DnaSP v6.12.03 software [22]. Median-joining (MJ) network analysis [23] was performed using NETWORK ver. 10.2.0.0 (http://www.fluxus-engineering.com), with transversion being weighted twice as transition in the analysis, and highly variable characters were down-weighted to resolve high-dimensional cubes and large network cycles. The analysis was conducted using an epsilon value of 70 to ensure a full median network based on the range of genetic distances in the dataset. Maximum parsimony (MP) calculation [24] was applied to generate the shortest network tree.

Phylogenetic analysis was performed using RAxML v.8.2.12 [25] through raxmlGUI 2.0.13 [26]. Codon position partitioning and the substitution model (GTR + G) were selected based on results from PartitionFinder 2 v.2.1.1 [27].

## 3. Results

The Korean samples of *Lymantria dispar asiatica* (*n* = 84; *L* = 657 bp) showed low nucleotide diversity (*π* = 0.00159 ± 0.00022 SE) but high haplotype diversity (*Hd* = 0.660 ± 0.053 SD). Neutrality tests were significantly negative (Tajima’s *D* = −1.84565, *p* < 0.05; Fu and Li’s D* = −3.11568, *p* < 0.05; Fu and Li’s F* = −3.16555, *p* < 0.02).

**Figure 2:**
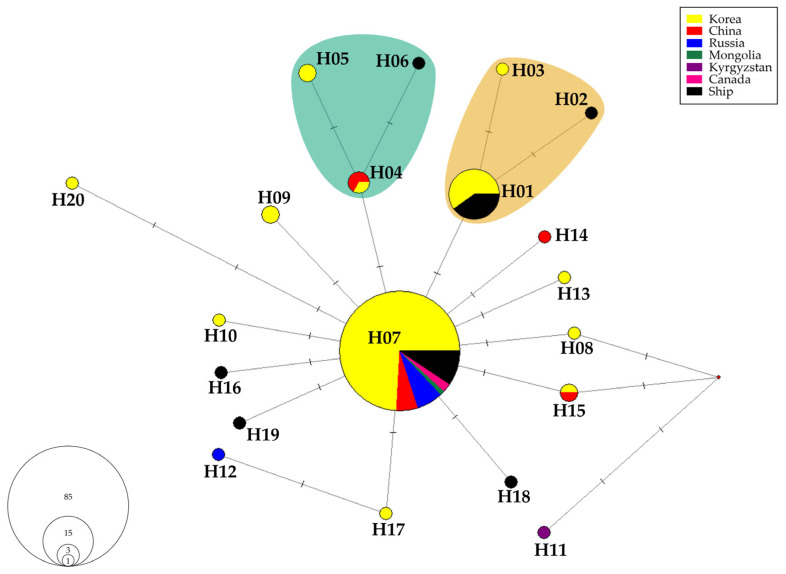
MJ network of *Lymantria dispar asiatica* haplotypes in Korea and adjacent countries. Haplotypes are color-coded by the country of collection. Mutational differences between haplotypes are indicated along the branches. The Southern haplogroup is shown as the orange cluster, and the Middle haplogroup is shown as the teal cluster.

The MJ analysis identified 20 haplotypes of *Lymantria dispar asiatica* (Figure 2 and Table 2; see also Supplementary Table S2 for detailed information). Among these, 12 haplotypes were found within Korean sequences. The most common haplotype, H07, appeared in 85 sequences from all countries, except Kyrgyzstan. This haplotype spanned 21 regions across South Korea, representing the entire country. The haplotype from Kyrgyzstan was the most genetically distant from H07, differing by four characters. Within the Korean sequences, the most distant haplotype was H20, which differed by three characters from H07.

**Figure 3:**
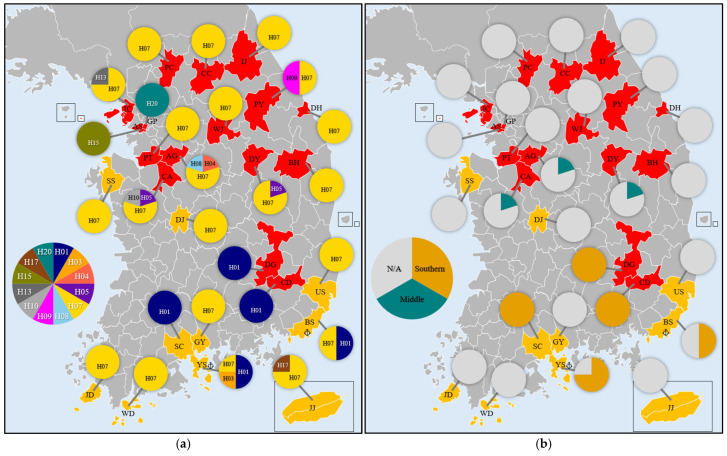
Distribution of haplotypes and haplogroups of *Lymantria dispar asiatica* in Korea. (**a**) Haplotype distribution: each color represents a distinct haplotype. (**b**) Haplogroup distribution: orange indicates the Southern haplogroup and teal indicates the Middle haplogroup. Anchor marks indicate harbors.

The second most frequent haplotype, H01, was identified in 15 sequences from Korea and ships. H01 was present in the southern regions of Korea, including Busan, Cheongdo, Daegu, Suncheon, and Yeosu (Figure 3). H01 differed by one character from haplotypes H02 and H03, which were collected from the southern regions. These three closely related haplotypes were designated as the Southern haplogroup.

Haplotypes H04 and H05, collected from similar latitudes in Korea, differed by one character. Haplotype H06 also differed from H04 by one character (Figure 3). These three haplotypes (H04, H05, and H06) were classified as the Middle haplogroup.

To further assess the clustering of haplotypes, ML analysis was conducted. The resulting ML tree showed distinct clustering of the two haplogroups, although the backbone of the tree was not supported (Supplementary Figure S1).

## 4. Discussion

The results revealed 12 haplotypes from Korea, highlighting the diverse populations of *Lymantria dispar asiatica* in the region. Despite this diversity, the genetic variation within Korean samples appeared low. Most sequences were grouped under a single haplotype, H07, while the remaining sequences were distinct, exhibiting several differences from H07. To minimize bias from the sample size, we included a maximum of five sequences per region. Nevertheless, haplotype H07 was present across all regions of Korea and in neighboring countries. This suggests that multiple invasive populations may have been introduced into Korea through human activities such as international trade and travel.

The low genetic diversity observed in Korean *L. dispar asiatica* populations may be attributed to the outbreak of this species in 2020. Due to their flight capability and broad range of host plants, these moths rapidly spread across the Korean Peninsula. This extensive dispersal likely contributed to the reduced genetic diversity within the population. This interpretation is supported by the pattern of low nucleotide diversity, high haplotype diversity, significantly negative neutrality tests (Tajima’s *D*, Fu and Li’s D*, and F*), and a star-shaped haplotype network.

The Southern haplogroup primarily consisted of samples collected from ships and harbors, suggesting a potential origin from invasive haplotypes. In contrast, the Middle haplogroup comprised sequences from Korea’s central region and the Shandong region of China. The proximity of Shandong to Korea, with its peninsula extending into the Yellow Sea, suggests a shared population across these regions. This geographical connection suggests that L. dispar asiatica populations in central Korea may be genetically similar to those in Shandong, regardless of their origin.

A previous study by Kang et al. [15] investigated the genetic structure of this species in Korea and neighboring countries, identifying three haplogroups: Group 1 (Korean in-land and adjacent areas), Group 2 (southern Korea), and Group 3 (Hokkaido). Although the specific genetic differences between groups were not detailed, the distinction between southern and inland haplotypes was evident. The presence of the southern haplotype group aligns with the Southern haplogroup identified in our study.

Haplotype H11, originating from Kyrgyzstan, exhibited the greatest genetic distance from the dominant H07 haplotype. Zahiri et al. [17] described three major lineages of *Lymantria dispar*, irrespective of subspecies: the “Europe + Central Asia lineage,” the “East Asia + Japan lineage,” and the “Transcaucasia lineage.” Although our study focused exclusively on *L. d. asiatica*, the sampling range spanned from Central to East Asia. The genetic divergence observed between H11 and the other haplotypes supports the lineage distinctions proposed by Zahiri et al. [17].

## 5. Conclusions

This study characterized the genetic diversity of *Lymantria dispar asiatica* populations in Korea. The results revealed low nucleotide diversity but high haplotype diversity, suggesting that these populations have expanded recently and have been affected by alien populations. These findings emphasize the need for strict monitoring at ports to prevent the introduction of invasive pests and the importance of forest pest management. This study was limited to mitochondrial COI fragments. Future studies should include nuclear markers like microsatellites or SNPs to achieve higher resolution. In addition, broader sampling across East Asia and temporal monitoring of haplotypes should be conducted to better understand and track ongoing changes in haplotype composition.

## Figures and Tables

**Table 1:** Geographic origin and number of samples analyzed.

Country	Region	Locality	Number of Samples	Reference
Canada	British Columbia	-	2	deWaard et al. (2010) [13]
China	Beijing	-	3	deWaard et al. (2010) [13]
China	Hebei	-	3	deWaard et al. (2010) [13]
China	Liaoning	-	1	deWaard et al. (2010) [13]
China	Shandong	-	2	deWaard et al. (2010) [13]
Korea	BS	Harbor	2	Kang et al. (2015) [18]
Korea	CB	Danyang-gun	5	this study
Korea	CN	Cheonan-si	5	this study
Korea	CN	Mt. Geumgangsan, Seosan-si	1	Kang et al. (2015) [18]
Korea	DG	-	2	this study
Korea	DJ	-	5	this study
Korea	GB	Bonghwa-gun	5	this study
Korea	GB	Cheongdo-gun	1	this study
Korea	GG	Anseong-si	5	this study
Korea	GG	Is. Daebudo, Ansan-si	1	Kang et al. (2015) [18]
Korea	GG	Mt. Surisan, Gunpo-si	1	Kang et al. (2015) [18]
Korea	GG	Pocheon-si	5	this study
Korea	GG	Pyeongtaek-si	4	this study
Korea	GW	-	2	Stewart et al. (2016) [19]
Korea	GW	Chuncheon-si	3	this study
Korea	GW	Donghae-si	2	this study
Korea	GW	Inje-gun	2	this study
Korea	GW	Mt. Odaesan, Pyeongchang-gun	1	Kang et al. (2015) [18]
Korea	GW	Pyeongchang-gun	3	this study
Korea	GW	Wonju-si	5	this study
Korea	IC	Is. Yeonpyeongdo	3	Kang et al. (2015) [18]
Korea	IC	Mt. Horyonggoksan, Is. Muuido	1	Kang et al. (2015) [18]
Korea	JJ	Mt. Hallasan	4	Kang et al. (2015) [18]
Korea	JN	Gwangyang-si	5	this study
Korea	JN	Harbor, Yeosu-si	4	Kang et al. (2015) [18]
Korea	JN	Jindo-gun	1	Kang et al. (2015) [18]
Korea	JN	Suncheon-si	3	this study
Korea	JN	Wando-gun	2	Kang et al. (2015) [18]
Korea	US	-	1	this study
Kyrgyzstan	Jalal-Abad	Toktogul	1	deWaard et al. (2010) [13]
Mongolia	Ulaanbaatar	-	1	deWaard et al. (2010) [13]
Russia	-	-	1	Stewart et al. (2016) [19]
Russia	Primorsky Kray	-	5	deWaard et al. (2010) [13]
Russia	Primorsky Kray	Vladivostok	1	Stewart et al. (2016) [19]
Ship	-	China → Korea (Gunsan-si, JB)	5	Kang et al. (2015) [18]
Ship	-	China → Korea (Yeongam-gun, JN)	3	Kang et al. (2015) [18]
Ship	-	N/A → Korea (Gwangyang-si, JN)	1	Kang et al. (2015) [18]
Ship	-	N/A → Korea (Incheon)	5	Kang et al. (2015) [18]
Ship	-	The Americas → Russia → Korea (Ulsan)	5	Kang et al. (2015) [18]

**Table 2:** Haplogroups and haplotypes of *Lymantria dispar asiatica,* with their distribution.

Haplogroup	Haplotype	Country	Number of Samples	Distribution
Southern	H01	Korea	9	BS *, CD, DG, SC, YS *
Southern	H01	Ship	6	-
Southern	H02	Ship	1	-
Southern	H03	Korea	1	YS *
Middle	H04	China	2	Shandong
Middle	H04	Korea	1	AG
Middle	H05	Korea	2	CA, DY
Middle	H06	Ship	1	-
	H07	Canada	2	British Columbia
	H07	China	5	Beijing, Hebei
	H07	Korea	63	AG, BH, BS *, CA, CC, DH, DJ, DY, GW, GY, IC, IJ, JD, JJ, PC, PT, PY, SS, US, WD, WJ, YS *
	H07	Mongolia	1	Ulaanba-atar
	H07	Russia	6	Primorsky Kray
	H07	Ship	8	-
	H08	Korea	1	AG
	H09	Korea	2	PY
	H10	Korea	1	CA
	H11	Kyrgyzstan	1	Toktogul
	H12	Russia	1	-
	H13	Korea	1	IC
	H14	China	1	Beijing
	H15	China	1	Liaoning
	H15	Korea	1	AS
	H16	Ship	1	-
	H17	Korea	1	JJ
	H18	Ship	1	-
	H19	Ship	1	-
	H20	Korea	1	GP

The asterisks (*) indicate samples collected at harbors.

## Data Availability

All sequences used in this study are available in NCBI GenBank.

## Supplementary Materials

The following supporting information can be downloaded at https://www.mdpi.com/article/10.3390/insects16090958/s1: Figure S1: ML tree of *Lymantria dispar asiatica*; Table S1: Sample information and accession numbers of *Lymantria dispar asiatica*; Table S2: Designation of haplogroups and haplotypes in *Lymantria dispar asiatica*.

## Abbreviations

The following abbreviations are used in this manuscript:

AG	Anseong-si
AS	Ansan-si
BH	Bonghwa-gun
BS	Busan
CA	Cheonan-si
CB	Chungcheongbuk-do
CC	Chuncheon-si
CD	Cheongdo-gun
CN	Chungcheongnam-do
DG	Daegu
DH	Donghae-si
DJ	Daejeon
DY	Danyang-gun
GB	Gyeongsangbuk-do
GG	Gyeonggi-do
GP	Gunpo-si
GW	Gangwon-do
GY	Gwangyang-si
IC	Incheon
IJ	Inje-gun
JD	Jindo-gun
JJ	Jeju island
JN	Jeollanam-do
PC	Pocheon-si
PT	Pyeongtaek-si
PY	Pyeongchang-gun
SC	Suncheon-si
SS	Seosan-si
US	Ulsan
WD	Wando-gun
WJ	Wonju-si
YS	Yeosu-si
