# Supplementary material for: The Genetic Diversity of the Asian Spongy Moth, Lymantria dispar asiatica Vnukovskii (Lepidoptera: Erebidae), in Korea Based on Mitochondrial COI Analysis
Source: Insects. 2025 Sep 11;16(9):958. doi: 10.3390/insects16090958 (PMC12470964; doi:10.3390/insects16090958)

Table S1. Sample information and accession numbers of *Lymantria dispar asiatica*.

Sequence No.	Genus	Species	Subspecies	Country	Collecting site	Accession No.	Reference
CAN001	Lymantria	dispar	asiatica	Canada	British Columbia	HM775694	deWaard et al. (2010)
CAN002	Lymantria	dispar	asiatica	Canada	British Columbia	HM775695	deWaard et al. (2010)
CHN001	Lymantria	dispar	asiatica	China	Beijing	HM775690	deWaard et al. (2010)
CHN002	Lymantria	dispar	asiatica	China	Beijing	HM775691	deWaard et al. (2010)
CHN003	Lymantria	dispar	asiatica	China	Beijing	HM775692	deWaard et al. (2010)
CHN004	Lymantria	dispar	asiatica	China	Hebei	HM775687	deWaard et al. (2010)
CHN005	Lymantria	dispar	asiatica	China	Hebei	HM775688	deWaard et al. (2010)
CHN006	Lymantria	dispar	asiatica	China	Hebei	HM775689	deWaard et al. (2010)
CHN007	Lymantria	dispar	asiatica	China	Liaoning	HM775686	deWaard et al. (2010)
CHN008	Lymantria	dispar	asiatica	China	Shandong	HM775684	deWaard et al. (2010)
CHN009	Lymantria	dispar	asiatica	China	Shandong	HM775685	deWaard et al. (2010)
KOR001	Lymantria	dispar	asiatica	Korea	Anseong-si, GG	PV186653	this study
KOR002	Lymantria	dispar	asiatica	Korea	Anseong-si, GG	PV186654	this study
KOR003	Lymantria	dispar	asiatica	Korea	Anseong-si, GG	PV186655	this study
KOR004	Lymantria	dispar	asiatica	Korea	Anseong-si, GG	PV186656	this study
KOR005	Lymantria	dispar	asiatica	Korea	Anseong-si, GG	PV186657	this study
KOR006	Lymantria	dispar	asiatica	Korea	Bonghwa-gun, GB	PV186658	this study
KOR007	Lymantria	dispar	asiatica	Korea	Bonghwa-gun, GB	PV186659	this study
KOR008	Lymantria	dispar	asiatica	Korea	Bonghwa-gun, GB	PV186660	this study
KOR009	Lymantria	dispar	asiatica	Korea	Bonghwa-gun, GB	PV186661	this study
KOR010	Lymantria	dispar	asiatica	Korea	Bonghwa-gun, GB	PV186662	this study
KOR011	Lymantria	dispar	asiatica	Korea	Cheonan-si, CN	PV186663	this study
KOR012	Lymantria	dispar	asiatica	Korea	Cheonan-si, CN	PV186664	this study
KOR013	Lymantria	dispar	asiatica	Korea	Cheonan-si, CN	PV186665	this study

Sequence No.	Genus	Species	Subspecies	Country	Collecting site	Accession No.	Reference
KOR014	Lymantria	dispar	asiatica	Korea	Cheonan-si, CN	PV186666	this study
KOR015	Lymantria	dispar	asiatica	Korea	Cheonan-si, CN	PV186667	this study
KOR016	Lymantria	dispar	asiatica	Korea	Daejeon	PV186668	this study
KOR017	Lymantria	dispar	asiatica	Korea	Daejeon	PV186669	this study
KOR018	Lymantria	dispar	asiatica	Korea	Daejeon	PV186670	this study
KOR019	Lymantria	dispar	asiatica	Korea	Daejeon	PV186671	this study
KOR020	Lymantria	dispar	asiatica	Korea	Daejeon	PV186672	this study
KOR021	Lymantria	dispar	asiatica	Korea	Danyang-gun, CB	PV186673	this study
KOR022	Lymantria	dispar	asiatica	Korea	Danyang-gun, CB	PV186674	this study
KOR023	Lymantria	dispar	asiatica	Korea	Danyang-gun, CB	PV186675	this study
KOR024	Lymantria	dispar	asiatica	Korea	Danyang-gun, CB	PV186676	this study
KOR025	Lymantria	dispar	asiatica	Korea	Danyang-gun, CB	PV186677	this study
KOR026	Lymantria	dispar	asiatica	Korea	Donghae-si, GW	PV186678	this study
KOR027	Lymantria	dispar	asiatica	Korea	Donghae-si, GW	PV186679	this study
KOR028	Lymantria	dispar	asiatica	Korea	GW	KX436543	Stewart et al. (2016)
KOR029	Lymantria	dispar	asiatica	Korea	GW	KX436544	Stewart et al. (2016)
KOR030	Lymantria	dispar	asiatica	Korea	Gwangyang-si, JN	PV186680	this study
KOR031	Lymantria	dispar	asiatica	Korea	Gwangyang-si, JN	PV186681	this study
KOR032	Lymantria	dispar	asiatica	Korea	Gwangyang-si, JN	PV186682	this study
KOR033	Lymantria	dispar	asiatica	Korea	Gwangyang-si, JN	PV186683	this study
KOR034	Lymantria	dispar	asiatica	Korea	Gwangyang-si, JN	PV186684	this study
KOR035	Lymantria	dispar	asiatica	Korea	Harbor, Busan	KF746208	Kang et al. (2015)
KOR036	Lymantria	dispar	asiatica	Korea	Harbor, Busan	KF746218	Kang et al. (2015)
KOR037	Lymantria	dispar	asiatica	Korea	Harbor, Yeosu-si, JN	KF746201	Kang et al. (2015)
KOR038	Lymantria	dispar	asiatica	Korea	Harbor, Yeosu-si, JN	KF746210	Kang et al. (2015)
KOR039	Lymantria	dispar	asiatica	Korea	Harbor, Yeosu-si, JN	KF746211	Kang et al. (2015)

Sequence No.	Genus	Species	Subspecies	Country	Collecting site	Accession No.	Reference
KOR040	Lymantria	dispar	asiatica	Korea	Harbor, Yeosu-si, JN	KF746225	Kang et al. (2015)
KOR041	Lymantria	dispar	asiatica	Korea	Is. Daebudo, Ansan-si, GG	KF746252	Kang et al. (2015)
KOR042	Lymantria	dispar	asiatica	Korea	Is. Yeonpyeongdo, Incheon	KF746219	Kang et al. (2015)
KOR043	Lymantria	dispar	asiatica	Korea	Is. Yeonpyeongdo, Incheon	KF746222	Kang et al. (2015)
KOR044	Lymantria	dispar	asiatica	Korea	Is. Yeonpyeongdo, Incheon	KF746235	Kang et al. (2015)
KOR045	Lymantria	dispar	asiatica	Korea	Jindo-gun, JN	KF746240	Kang et al. (2015)
KOR046	Lymantria	dispar	asiatica	Korea	Mt. Geumgangsan, Seosan-si, CN	KF746224	Kang et al. (2015)
KOR047	Lymantria	dispar	asiatica	Korea	Mt. Hallasan, JJ	KF746220	Kang et al. (2015)
KOR048	Lymantria	dispar	asiatica	Korea	Mt. Hallasan, JJ	KF746223	Kang et al. (2015)
KOR049	Lymantria	dispar	asiatica	Korea	Mt. Hallasan, JJ	KF746233	Kang et al. (2015)
KOR050	Lymantria	dispar	asiatica	Korea	Mt. Hallasan, JJ	KF746234	Kang et al. (2015)
KOR051	Lymantria	dispar	asiatica	Korea	Mt. Horyonggoksan, Is. Muuido, Incheon	KF746254	Kang et al. (2015)
KOR052	Lymantria	dispar	asiatica	Korea	Mt. Odaesan, Pyeongchang-gun, GW	KF746221	Kang et al. (2015)
KOR053	Lymantria	dispar	asiatica	Korea	Mt. Surisan, Gunpo-si, GG	KF746247	Kang et al. (2015)
KOR054	Lymantria	dispar	asiatica	Korea	Pyeongchang-gun, GW	PV186685	this study
KOR055	Lymantria	dispar	asiatica	Korea	Pyeongchang-gun, GW	PV186686	this study
KOR056	Lymantria	dispar	asiatica	Korea	Pyeongchang-gun, GW	PV186687	this study
KOR057	Lymantria	dispar	asiatica	Korea	Pyeongtaek-si, GG	PV186688	this study
KOR058	Lymantria	dispar	asiatica	Korea	Pyeongtaek-si, GG	PV186689	this study
KOR059	Lymantria	dispar	asiatica	Korea	Pyeongtaek-si, GG	PV186690	this study
KOR060	Lymantria	dispar	asiatica	Korea	Pyeongtaek-si, GG	PV186691	this study
KOR061	Lymantria	dispar	asiatica	Korea	Suncheon-si, JN	PV186692	this study
KOR062	Lymantria	dispar	asiatica	Korea	Suncheon-si, JN	PV186693	this study
KOR063	Lymantria	dispar	asiatica	Korea	Suncheon-si, JN	PV186694	this study
KOR064	Lymantria	dispar	asiatica	Korea	Ulsan	PV186695	this study
KOR065	Lymantria	dispar	asiatica	Korea	Wando-gun, JN	KF746236	Kang et al. (2015)

Sequence No.	Genus	Species	Subspecies	Country	Collecting site	Accession No.	Reference
KOR066	Lymantria	dispar	asiatica	Korea	Wando-gun, JN	KF746237	Kang et al. (2015)
KOR067	Lymantria	dispar	asiatica	Korea	Wonju-si, GW	PV186696	this study
KOR068	Lymantria	dispar	asiatica	Korea	Wonju-si, GW	PV186697	this study
KOR069	Lymantria	dispar	asiatica	Korea	Wonju-si, GW	PV186698	this study
KOR070	Lymantria	dispar	asiatica	Korea	Wonju-si, GW	PV186699	this study
KOR071	Lymantria	dispar	asiatica	Korea	Wonju-si, GW	PV186700	this study
KOR072	Lymantria	dispar	asiatica	Korea	Chuncheon-si, GW	PV983671	this study
KOR073	Lymantria	dispar	asiatica	Korea	Chuncheon-si, GW	PV983672	this study
KOR074	Lymantria	dispar	asiatica	Korea	Chuncheon-si, GW	PV983673	this study
KOR075	Lymantria	dispar	asiatica	Korea	Inje-gun, GW	PV983674	this study
KOR076	Lymantria	dispar	asiatica	Korea	Inje-gun, GW	PV983675	this study
KOR077	Lymantria	dispar	asiatica	Korea	Daegu	PV983676	this study
KOR078	Lymantria	dispar	asiatica	Korea	Daegu	PV983677	this study
KOR079	Lymantria	dispar	asiatica	Korea	Cheongdo-gun, GB	PV983678	this study
KOR080	Lymantria	dispar	asiatica	Korea	Pocheon-si, GG	PV983679	this study
KOR081	Lymantria	dispar	asiatica	Korea	Pocheon-si, GG	PV983680	this study
KOR082	Lymantria	dispar	asiatica	Korea	Pocheon-si, GG	PV983681	this study
KOR083	Lymantria	dispar	asiatica	Korea	Pocheon-si, GG	PV983682	this study
KOR084	Lymantria	dispar	asiatica	Korea	Pocheon-si, GG	PV983683	this study
KYR001	Lymantria	dispar	asiatica	Kyrgyzstan	Toktogul	HM775701	deWaard et al. (2010)
MGR001	Lymantria	dispar	asiatica	Mongolia	Ulaanba-atar	HM775708	deWaard et al. (2010)
RUS001	Lymantria	dispar	asiatica	Russia	-	KX436538	Stewart et al. (2016)
RUS002	Lymantria	dispar	asiatica	Russia	Primorsky Kray	HM775582	deWaard et al. (2010)
RUS003	Lymantria	dispar	asiatica	Russia	Primorsky Kray	HM775583	deWaard et al. (2010)
RUS004	Lymantria	dispar	asiatica	Russia	Primorsky Kray	HM775584	deWaard et al. (2010)
RUS005	Lymantria	dispar	asiatica	Russia	Primorsky Kray	HM775618	deWaard et al. (2010)

Sequence No.	Genus	Species	Subspecies	Country	Collecting site	Accession No.	Reference
RUS006	Lymantria	dispar	asiatica	Russia	Primorsky Kray	KX436541	Stewart et al. (2016)
RUS007	Lymantria	dispar	asiatica	Russia	Vladivostok, Primorsky Kray	KX436542	Stewart et al. (2016)
SHP001	Lymantria	dispar	asiatica	Ship	China → Korea (Gunsan-si, JB)	KF746214	Kang et al. (2015)
SHP002	Lymantria	dispar	asiatica	Ship	China → Korea (Gunsan-si, JB)	KF746215	Kang et al. (2015)
SHP003	Lymantria	dispar	asiatica	Ship	China → Korea (Gunsan-si, JB)	KF746216	Kang et al. (2015)
SHP004	Lymantria	dispar	asiatica	Ship	China → Korea (Gunsan-si, JB)	KF746231	Kang et al. (2015)
SHP005	Lymantria	dispar	asiatica	Ship	China → Korea (Gunsan-si, JB)	KF746246	Kang et al. (2015)
SHP006	Lymantria	dispar	asiatica	Ship	China → Korea (Yeongam-gun, JN)	KF746202	Kang et al. (2015)
SHP007	Lymantria	dispar	asiatica	Ship	China → Korea (Yeongam-gun, JN)	KF746204	Kang et al. (2015)
SHP008	Lymantria	dispar	asiatica	Ship	China → Korea (Yeongam-gun, JN)	KF746228	Kang et al. (2015)
SHP009	Lymantria	dispar	asiatica	Ship	N/A → Korea (Gwangyang-si, JN)	KF746209	Kang et al. (2015)
SHP010	Lymantria	dispar	asiatica	Ship	N/A → Korea (Incheon)	KF746205	Kang et al. (2015)
SHP011	Lymantria	dispar	asiatica	Ship	N/A → Korea (Incheon)	KF746206	Kang et al. (2015)
SHP012	Lymantria	dispar	asiatica	Ship	N/A → Korea (Incheon)	KF746207	Kang et al. (2015)
SHP013	Lymantria	dispar	asiatica	Ship	N/A → Korea (Incheon)	KF746217	Kang et al. (2015)
SHP014	Lymantria	dispar	asiatica	Ship	N/A → Korea (Incheon)	KF746226	Kang et al. (2015)
SHP015	Lymantria	dispar	asiatica	Ship	The Americas → Russia → Korea (Ulsan)	KF746203	Kang et al. (2015)
SHP016	Lymantria	dispar	asiatica	Ship	The Americas → Russia → Korea (Ulsan)	KF746212	Kang et al. (2015)
SHP017	Lymantria	dispar	asiatica	Ship	The Americas → Russia → Korea (Ulsan)	KF746213	Kang et al. (2015)
SHP018	Lymantria	dispar	asiatica	Ship	The Americas → Russia → Korea (Ulsan)	KF746244	Kang et al. (2015)
SHP019	Lymantria	dispar	asiatica	Ship	The Americas → Russia → Korea (Ulsan)	KF746249	Kang et al. (2015)

Table S2. Designation of haplogroups and haplotypes in *Lymantria dispar asiatica*.

Haplogroup	Haplotype	Sequence No.	Country	Collecting site
Southern	H01	KOR035	Korea	Harbor, Busan
Southern	H01	KOR038	Korea	Harbor, Yeosu-si, JN
Southern	H01	KOR039	Korea	Harbor, Yeosu-si, JN
Southern	H01	KOR061	Korea	Suncheon-si, JN
Southern	H01	KOR062	Korea	Suncheon-si, JN
Southern	H01	KOR063	Korea	Suncheon-si, JN
Southern	H01	KOR077	Korea	Daegu
Southern	H01	KOR078	Korea	Daegu
Southern	H01	KOR079	Korea	Cheongdo-gun, GB
Southern	H01	SHP007	Ship	China → Korea (Yeongam-gun, JN)
Southern	H01	SHP009	Ship	N/A → Korea (Gwangyang-si, JN)
Southern	H01	SHP010	Ship	N/A → Korea (Incheon)
Southern	H01	SHP011	Ship	N/A → Korea (Incheon)
Southern	H01	SHP012	Ship	N/A → Korea (Incheon)
Southern	H01	SHP015	Ship	The Americas → Russia → Korea (Ulsan)
Southern	H02	SHP006	Ship	China → Korea (Yeongam-gun, JN)
Southern	H03	KOR037	Korea	Harbor, Yeosu-si, JN
Middle	H04	CHN008	China	Shandong
Middle	H04	CHN009	China	Shandong
Middle	H04	KOR004	Korea	Anseong-si, GG
Middle	H05	KOR015	Korea	Cheonan-si, CN
Middle	H05	KOR025	Korea	Danyang-gun, CB
Middle	H06	SHP019	Ship	The Americas → Russia → Korea (Ulsan)
	H07	CAN001	Canada	British Columbia
	H07	CAN002	Canada	British Columbia
	H07	CHN001	China	Beijing
	H07	CHN003	China	Beijing
	H07	CHN004	China	Hebei
	H07	CHN005	China	Hebei
	H07	CHN006	China	Hebei
	H07	KOR001	Korea	Anseong-si, GG
	H07	KOR002	Korea	Anseong-si, GG
	H07	KOR003	Korea	Anseong-si, GG
	H07	KOR006	Korea	Bonghwa-gun, GB
	H07	KOR007	Korea	Bonghwa-gun, GB
	H07	KOR008	Korea	Bonghwa-gun, GB
	H07	KOR009	Korea	Bonghwa-gun, GB
	H07	KOR010	Korea	Bonghwa-gun, GB

Haplogroup	Haplotype	Sequence No.	Country	Collecting site
	H07	KOR012	Korea	Cheonan-si, CN
	H07	KOR013	Korea	Cheonan-si, CN
	H07	KOR014	Korea	Cheonan-si, CN
	H07	KOR016	Korea	Daejeon
	H07	KOR017	Korea	Daejeon
	H07	KOR018	Korea	Daejeon
	H07	KOR019	Korea	Daejeon
	H07	KOR020	Korea	Daejeon
	H07	KOR021	Korea	Danyang-gun, CB
	H07	KOR022	Korea	Danyang-gun, CB
	H07	KOR023	Korea	Danyang-gun, CB
	H07	KOR024	Korea	Danyang-gun, CB
	H07	KOR026	Korea	Donghae-si, GW
	H07	KOR027	Korea	Donghae-si, GW
	H07	KOR028	Korea	GW
	H07	KOR029	Korea	GW
	H07	KOR030	Korea	Gwangyang-si, JN
	H07	KOR031	Korea	Gwangyang-si, JN
	H07	KOR032	Korea	Gwangyang-si, JN
	H07	KOR033	Korea	Gwangyang-si, JN
	H07	KOR034	Korea	Gwangyang-si, JN
	H07	KOR036	Korea	Harbor, Busan
	H07	KOR040	Korea	Harbor, Yeosu-si, JN
	H07	KOR042	Korea	Is. Yeonpyeongdo, Incheon
	H07	KOR043	Korea	Is. Yeonpyeongdo, Incheon
	H07	KOR044	Korea	Is. Yeonpyeongdo, Incheon
	H07	KOR045	Korea	Jindo-gun, JN
	H07	KOR046	Korea	Mt. Geumgangsan, Seosan-si, CN
	H07	KOR047	Korea	Mt. Hallasan, JJ
	H07	KOR048	Korea	Mt. Hallasan, JJ
	H07	KOR050	Korea	Mt. Hallasan, JJ
	H07	KOR052	Korea	Mt. Odaesan, Pyeongchang-gun, GW
	H07	KOR054	Korea	Pyeongchang-gun, GW
	H07	KOR057	Korea	Pyeongtaek-si, GG
	H07	KOR058	Korea	Pyeongtaek-si, GG
	H07	KOR059	Korea	Pyeongtaek-si, GG
	H07	KOR060	Korea	Pyeongtaek-si, GG
	H07	KOR064	Korea	Ulsan
	H07	KOR065	Korea	Wando-gun, JN
	H07	KOR066	Korea	Wando-gun, JN

Haplogroup	Haplotype	Sequence No.	Country	Collecting site
	H07	KOR067	Korea	Wonju-si, GW
	H07	KOR068	Korea	Wonju-si, GW
	H07	KOR069	Korea	Wonju-si, GW
	H07	KOR070	Korea	Wonju-si, GW
	H07	KOR071	Korea	Wonju-si, GW
	H07	KOR072	Korea	Chuncheon-si, GW
	H07	KOR073	Korea	Chuncheon-si, GW
	H07	KOR074	Korea	Chuncheon-si, GW
	H07	KOR075	Korea	Inje-gun, GW
	H07	KOR076	Korea	Inje-gun, GW
	H07	KOR080	Korea	Pocheon-si, GG
	H07	KOR081	Korea	Pocheon-si, GG
	H07	KOR082	Korea	Pocheon-si, GG
	H07	KOR083	Korea	Pocheon-si, GG
	H07	KOR084	Korea	Pocheon-si, GG
	H07	MGR001	Mongolia	Ulaanba-atar
	H07	RUS002	Russia	Primorsky Kray
	H07	RUS003	Russia	Primorsky Kray
	H07	RUS004	Russia	Primorsky Kray
	H07	RUS005	Russia	Primorsky Kray
	H07	RUS006	Russia	Primorsky Kray
	H07	RUS007	Russia	Vladivostok, Primorsky Kray
	H07	SHP001	Ship	China → Korea (Gunsan-si, JB)
	H07	SHP002	Ship	China → Korea (Gunsan-si, JB)
	H07	SHP003	Ship	China → Korea (Gunsan-si, JB)
	H07	SHP008	Ship	China → Korea (Yeongam-gun, JN)
	H07	SHP013	Ship	N/A → Korea (Incheon)
	H07	SHP014	Ship	N/A → Korea (Incheon)
	H07	SHP016	Ship	The Americas → Russia → Korea (Ulsan)
	H07	SHP017	Ship	The Americas → Russia → Korea (Ulsan)
	H08	KOR005	Korea	Anseong-si, GG
	H09	KOR055	Korea	Pyeongchang-gun, GW
	H09	KOR056	Korea	Pyeongchang-gun, GW
	H10	KOR011	Korea	Cheonan-si, CN
	H11	KYR001	Kyrgyzstan	Toktogul
	H12	RUS001	Russia	-
	H13	KOR051	Korea	Mt. Horyonggoksan, Is. Muuido, Incheon
	H14	CHN002	China	Beijing
	H15	CHN007	China	Liaoning
	H15	KOR041	Korea	Is. Daebudo, Ansan-si, GG

Haplogroup	Haplotype	Sequence No.	Country	Collecting site
	H16	SHP004	Ship	China → Korea (Gunsan-si, JB)
	H17	KOR049	Korea	Mt. Hallasan, JJ
	H18	SHP018	Ship	The Americas → Russia → Korea (Ulsan)
	H19	SHP005	Ship	China → Korea (Gunsan-si, JB)
	H20	KOR053	Korea	Mt. Surisan, Gunpo-si, GG

Supplementary Figure S1. ML tree of *Lymantria dispar asiatica*. The Southern haplogroup is shown as the orange cluster, and the Middle haplogroup as the teal cluster.

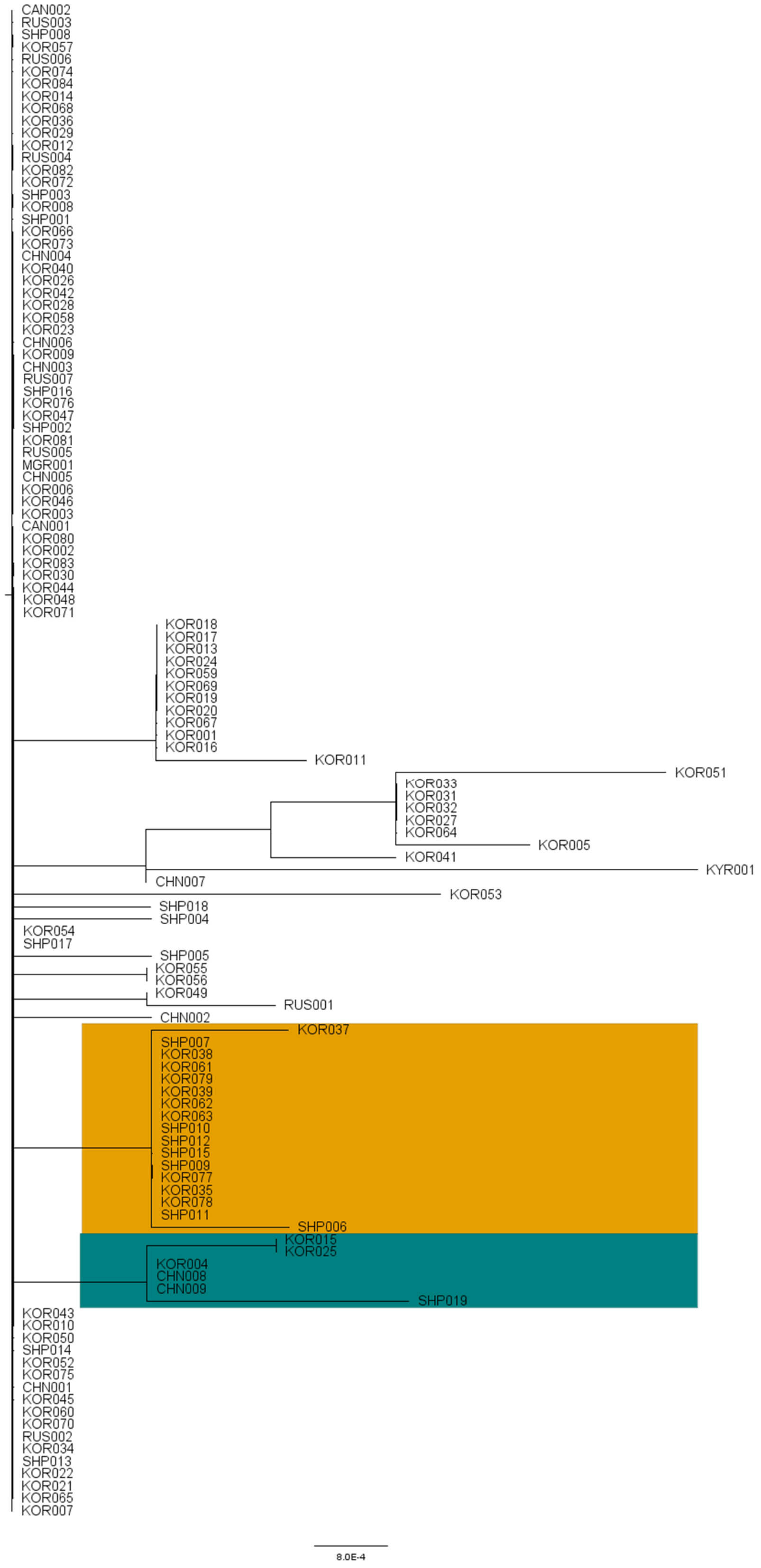

Supplement: Supplementary file 1 [file insects-16-00958-s001.zip › insects-3840794-supplementary.pdf]
